# Higher hair cortisol levels associated with previous cardiovascular events and cardiovascular risks in a large cross-sectional population study

**DOI:** 10.1186/s12872-024-04221-2

**Published:** 2024-10-04

**Authors:** Åshild Faresjö, Elvar Theodorsson, Andreas Stomby, Helena Quist, Michael P. Jones, Carl Johan Östgren, Per Dahlqvist, Tomas Faresjö

**Affiliations:** 1 Department of Health, Medicine and Caring Sciences, Social Medicine and Public Health, Linköping University, Linköping, Sweden; 2 Department of Biomedical and Clinical Science, Division of Clinical Chemistry and Pharmacology, Linköping University, Linköping, Sweden; 3 Department of Health, Medicine and Caring Sciences, General Practice, Linköping University, Linköping, Sweden; 4 School of Psychological Sciences, Macquarie University, Sydney, NSW Australia; 5 Centre of Medical Image Science and Visualization, CMIV, Linköping University, Linköping, Sweden; 6 Department of Public Health and Clinical Medicine, Umeå University, Umeå, Sweden

**Keywords:** Cardiovascular diseases, Cardiovascular risks, Cortisol, Hair, Stress

## Abstract

**Background:**

Stress is today a common feature of patients seeking medical care and a growing public health issue in society. A method has been developed to measure biological chronic stress by Hair Cortisol Concentrations (HCC). This biomarker, for chronic stress, captures information about cumulative cortisol levels over the course of several months. Long-term stress might be one of the factors contributing to the onset of cardiovascular conditions and also affecting different risk factors. The aim of this study was to analyse the association between Hair Cortisol Concentrations and previous cardiovascular diseases and cardiovascular risk factors.

**Methods:**

The method of measuring chronic stress by Hair Cortisol Concentration was applied in a large Swedish national observational cross-sectional study. A population-based random sample of *N* = 4,821 Swedish middle-aged men and women was analysed for hair cortisol levels in relation to diagnosed previous cardiovascular diseases and biologically measured cardiovascular risk factors.

**Results:**

Long-term stress, measured by hair cortisol, was significantly associated with the classical cardiovascular risk factors hypertension and high cholesterol, but not smoking. Those with elevated HCC levels also had a significantly increased pre-history of myocardial infarction, type 2 diabetes, atrial fibrillation and by-pass surgery, but not regarding stroke, angina pectoris or sleep apnoea. Higher HCC was significantly associated (*p* < 0.001) with Body mass index and waist circumference, but only for females. HCC was also associated with the risk markers leukocytes, and high-sensitivity CRP, indicating a possible linkage between HCC and inflammation and hypothetically also the bodily immune defense. No association was found between perceived stress and HCC.

**Conclusions:**

An overall conclusion of our results is that health care should put more emphasis on patients reporting that they have been exposed to long term stress. Altogether, these analyses of Hair cortisol levels in a large middle-aged population show that chronically elevated cortisol levels represent a relevant and significant factor associated with cardiovascular diseases and classical cardiovascular risk factors.

## Introduction

A stressful everyday life is typical for current societies and especially long-term stress exposure is an important cause of public health challenges. Stress symptoms are today among the most predominant features of patients seeking medical attention in primary health care [1]. Cortisol, synthesized and released from the adrenal cortex under the control of the hypothalamic-pituitary-adrenal axis (HPA-axis), is one of the major stress hormones, with profound effects on most organs. Long-term exposure to elevated cortisol levels could lead to an increased risk of cardiovascular disease and death [2]. Cortisol levels may be used as a biomarker of stress when measured in saliva, blood, or urine. However, since circulating cortisol has a short half-life, approximately 1.5 h, and varies considerably with circadian rhythm and brief stressors, these methods provide limited information about long-term cortisol secretion [3, 4]. In the last decade, there has been an increased use of hair cortisol concentrations (HCC) as an alternative biomarker for chronic stress, capturing information about cumulative cortisol levels over the course of several months [5–7].

The concentrations of cortisol deposited in the hair are retrospective reflections of the biologically active plasma concentrations of the hormone during the period of growth [8]. Scalp hair grows approximately 1 cm/month, enabling a timed retrospective evaluation of long-term cortisol levels using only one sample. Hormone analysis in hair has the additional advantage of circumventing difficulties in determining the biologically active fraction and being stable at room temperature for months and years facilitating storage and [4].

So far, research exploring possible associations between cortisol concentrations in hair and diseases has found significant correlations of higher HCC with e.g., mental disorders and chronic pain and experiences of serious life events [10–13]. The associations between a variety of psychosocial factors and HCC might also potentially be related to genetic factors [13]. However, whether HCC and psychological factors share genetic risk factors remains unclear [1]. In studies where both self-reported stress and hair cortisol concentrations (HCC) have been measured, no consistent associations have been evident [15]. High perceived stress does not necessarily correspond to elevated HCC, since HCC is related to long-term negative stress exposures rather than stress caused by daily hazels [9]. Increased HCC in healthy working middle-aged women in different professions has been associated with higher perceived stress, generally poorer health, and depressiveness [16]. Elevated levels of cortisol in hair, indicating long stress exposure, were found among those who suffered an acute myocardial infarction (MI) compared to a control group without MI [17, 18]. Higher cortisol in saliva or serum was shown to be associated with cardiovascular risk factors and a higher incidence of cardiovascular disease [19]. Elevated HCC levels have also been associated with diseases like obesity, type 2 diabetes, high Body Mass Index (BMI), and heart failure [20, 21]. The biological mechanisms how long-term stress measured as HCC might affect a variety of cardiovascular disorders, is yet to be studied [22]. That could yield the risk for heart failure or coronary artery disease severity in patients with non-ST segment elevation myocardial infarction or unstable angina pectoris [23].

Long-term stress might be one of the factors contributing to the onset of different cardiovascular conditions. Against this background, more large-scale population-based studies are needed to investigate the potential role of HCC in relation to common cardiovascular risk factors.

### Aim of the study

This study aimed to analyze the association between Hair Cortisol Concentrations, and previous cardiovascular diseases and cardiovascular risk factors, in a large Swedish middle-aged national observational cross-sectional study.

## Materials and methods

### Study design and participants

Data in the present study derive from The Swedish Cardio Pulmonary bioImage Study (SCAPIS) conducted during 2015–2018. This is a major prospective national observational cross-sectional study of a randomly selected sample of over 30,000 middle-aged persons (50–64 years) from the general population in Sweden [21, 24, 25]. The overall objective of the national SCAPIS study was to investigate cardiovascular disease mechanisms and possible new biomarkers [26]. The study participants in SCAPIS were recruited from six Swedish university hospitals [24].

An additional data collection within the national SCAPIS study was initiated at two SCAPIS sites, focusing on measuring the cortisol concentration in hair. For the total random sample of 7,564 participants and of these 4,921 individuals provided hair samples, of which 3,462 were from one site (Linköping) and 1,459 from the other site (Umeå) (a total of 1,712 males and 3,209 females), which was included in this report. Hair samples and cortisol measurement were unavailable in 34.9% (*n* = 2,643), mainly owing to short hair length, only a few participants with sufficient hair length were unwilling to provide hair samples. All hair samples were analyzed at the research laboratory of clinical chemistry at Linköping University Hospital in Sweden.

### Questionnaires

An extensive questionnaire covered background data on lifestyle, social and psychosocial factors as well as self-reported medication and diagnoses. Socioeconomic status was based on education and divided into low, medium, and high; smoking was divided into yes regular smoker vs. sometimes and no or stopped smoking. Age was divided into three age groups based on age at study inclusion: 50–54, 55–59, and 60–65 years. Marital status was divided into single/unmarried, married/cohabited, divorced/separated, and widow/widower. Ethnicity was categorized as Swedish, Nordic (Norway, Finland, Iceland, and Denmark), European, and outside Europe.

Physicians’ diagnoses of hypertension and elevated plasma cholesterol concentrations were reported and dichotomized into yes or no. Family history of myocardial infarction and stroke was defined as those reporting parental myocardial infarction or stroke before < 60 years of age. A question about self-reported stress was divided into a 3-grade scale of never stressed, sometimes stressed, or always stressed now and the last 5 years. Self-reports of physical activity for the last 12 months were divided into none, little activity, or regular physical activities.

### Anthropometric measures

Body mass index (BMI) was divided into underweight (BMI < 18.5), normal BMI (18.5–25), overweight (BMI > 25–30), and obese (BMI > 30), and waist circumference was divided into five grades: 60–79 cm, 80–87 cm, 88–94 cm, 95–101 cm, and 102–168 cm according to standard procedure.

### Risk factors and diseases

Data on diseases and risk factors were included as self-reports in the SCAPIS questionnaire [26]. These included reports of physicians’ previous diagnoses of myocardial infarction, angina pectoris, atrial fibrillation, heart failure, bypass surgery/PCI, stroke, type 2 diabetes, sleep apnea, high cholesterol levels, and hypertension. The laboratory blood measurements in this study were performed at two separate but synchronized standardized routine laboratories including leukocyte numbers and glucose and high-sensitivity C-reactive protein (Hs-CRP) levels, these were classified as being within or above the reference values. An open question about the use of glucocorticoid medication was also asked. In the total cohort, *n* = 478 participants stated the use of some kind of corticoids such as ointments/cream, inhalation, tablets, or a mixture of these. Participants reporting regular use of glucocorticoids based on ointments/cream (*n* = 100) had significantly (*p* = 0.001) higher HCC values and were omitted from further analysis since they could potentially represent a confounding factor. Females with glucocorticoid treatment (*n* = 255) (excluding those treated with ointments/cream) had a significantly higher HCC (*p* = 0.01) than those without glucocorticoid use. There was no statistical difference (*p* = 0.07) between males in this respect, see Table 1.

**Table 1:** Median HCC values for individuals treated with glucocorticoids compared to HCC values for those without glucocorticoids. Individuals with ointments/cream treatments *n* = 100 are deleted from the analysis

	No corticoid treatment. Median pg/mg (IQR)	Yes, corticoid treatment. Median pg/mg (IQR)	*p*-value
Females	24.04 (39.4)	40.45 (79.7)	0.01
Males	32.25 (52.5)	50.62 (117.5)	0.07

### Analysing cortisol levels in hair

Hair, with a length of 3 cm, was cut from the vertex posterior of the scalp as close to the skin as possible by trained staff, as recommended by the Society of Hair Testing (SoHT) [27]. Hair in this area has a regular rate of growth [28–30]. Hair color was categorized in connection with the laboratory analysis. The hair samples were stored in sample tubes at room temperature. A competitive radioimmunoassay (RIA) was used to extract and analyze cortisol levels, as described by Yang et al. [31, 32]. Briefly, the hair sample was cut into 3 cm pieces and measured with a ruler starting from the root. Samples weighing 5–7 mg were frozen for 2 min in liquid nitrogen and minced together with a steel ball in a Retch Cryo Mill (20 HZ) for 2 min. Methanol (1 ml) was added to each tube, and the samples were extracted overnight on a moving board. Then 0.8 ml of the methanol supernatant was pipetted off and lyophilized using a Savant Speed Vac Plus SC210A. The samples were dissolved in radioimmunoassay buffer and analyzed as described by Mörelius et al. [33]. The primary antibody used was a rabbit polyclonal antibody “Cortisol 3”, catalog number MBS535414 (MyBioSource, San Diego, USA). Its reactivity profile is as follows: cortisol 100%, prednisolone 37%, 11-deoxycortisol 5%, corticosterone 3%, and cortisone < 1%. The secondary solid-phase antibody was anti-rabbit Sac-Cel (AA-Sac1, ImmunoDiagnostic Systems Ltd, Boldon, England). Previous work has shown that hair samples of 5 mg or more are needed to achieve an inter-assay coefficient of variation below 8% for hair extraction and cortisol measurement [9, 11, 16]. The intra-assay coefficient of variation for the radioimmunoassay was 7% at 10 nmol/L.

There are no established reference values of HCC for middle-aged populations. However, we have defined tentative reference values based on our previous research on different populations. According to these, the tentative reference HCC concentrations in healthy adults are approximately between 17 mg/pg to 84 mg /pg. The same pattern is seen in this study population based on *n* = 3,209 female participants and *n* = 1,712 male participants, see Table 2.

**Table 2:** Hair cortisol levels were distributed into tentative reference values in the SCAPIS population divided by gender

Variables	Low < 16 mg/pg	Medium	Higher > 85 pg/mg	*p*-value
Gender	*n* %	*n* %	*n* %	
Male	320 19.0	998 59.3	366 21.7	< 0.001
Female	952 30.3	1639 52.2	546 17.4	
Total	1272 26.4	2637 54.7	912 18.9	

To evaluate whether high HCC is associated with previous diagnoses of CVD, diabetes Type 2, and classical CVD risk factors, the HCC values in the studied cohort were also divided into deciles. The highest decile had an HCC > 180 pg/mg (*n* = 436) compared with the rest of the cohort (*n* = 4,385), which had an HCC up to 179.9 pg/mg.

### Statistical analysis

Median cortisol values and interquartile range (IQR) were used to describe the distribution of HCC values. Hair cortisol is compared across demographic and lifestyle factors via analysis of variance (Tables 3 and 4) from which the overall test of equality of median is reported. Tests of normality were made with Shapiro-Wilk tests, this showed a non-normal distribution of HCC with a long right-hand tail in the distribution and therefore logarithmically transformed HCC was used in univariate and multivariate analyses. In Tables 5 and 6 hair cortisol was dichotomized into the highest deciles vs. the lower deciles and categorical measures are reported as counts and percentages analysed by Chi^2^ and its association with risk factors is evaluated via correlations made with Spearman´s coefficients and logaritmized cortisol values as well as unconditional logistic regression in which the odds ratio is reported, along with 95% confidence interval and p-value. The term “significant” always refers to statistical significance (*p* < 0.05). Multivariate logistic regression using a stepwise backward elimination procedure was used to identify statistically independent discriminators of those in the highest decile of hair cortisol from those in lower deciles (Tables 5 and 6). All analyses were performed using SPSS version 28.

**Table 3:** Characteristics of the SCAPIS population in relation to hair cortisol concentrations expressed as HCC medians for females and males (*N* = 4821)

	Females (*N* = 3137) (Median HCC 25.13)^b^			Males (*N* = 1684) (Median HCC 32.57)^b^		
Variables	Median (IQR) ^a^	*n*	*p*-value	Median (IQR) ^a^	*n*	*p*-value
**Age-groups**			0.14			0.07
50–54 years	24.08 (38.2)	1100		31.48 (52.5)	577	
55–59 years	25.71 (40.8)	1011		32.39 (64.3)	548	
60–65 years	26.12 (47.4)	1026		**36.00** (59.3) ^b^	559	
**Education**:			0.92			0.34
Low	23.60 (36.1)	234		32.24 (51.5)	146	
Medium	25.25 (43.1)	1398		33.53 (56.7)	810	
High	25.09 (41.9)	1433		31.57 (54.3)	673	
**Civil status**			0.20			0.58
Singel/unmarried	25.89 (57.3)	299		32.65 (47.7)	190	
Married/cohabited	24.82 (41.1)	2482		32.98 (57.6)	1292	
Divorced/separated	25.35 (39.8)	299		29.70 (51.6)	121	
Widow/widower	26.43 (37.8)	71		**50.59** (78.7) ^b^	20	0.21^d^
**Ethnicity**			0.56			0.86
Swedish	24.62 (42.7)	2940		32.45 (53.9)	1612	
Nordic	**36.23** (48.3) *	75	0.01	**71.35** (115.5)^b^	19	
European	26.90 (34.3)	59		29.22 (33.5)	50	
Outside Europe	**31.06** (39.5)^b^	63	0.10	**39.59** (64.4)^b^	31	
**Hair color**			0.56			0.77
Blond/grey	27.44 (49.2)^b^	1991		34.75 (59.3)	1202	
Brown /black/red	26.03 (40.6)	705		**36.53** (54.2)^b^	840	

a) IQR measure dispersion, between 25th percentile and 75th among HCC value in each variable,

b) The median in bold expresses the median above the general median for both sexes

c)*Significant differences. d) P-value for widower

**Table 4:** Cardiovascular risk factors in relation to HCC among the SCAPIS population for females and males

	Females (*N* = 3137) (Median HCC 25.13)^c^			Males (*N* = 1684) (Median HCC 32.57)^c^		
Variables	Median (IQR) ^a^	*n*	*p*-value	Median (IQR) ^b^	*n*	*p*-value
**BMI**			< 0.001			0.38
Underweigth	**16.66** (14.9)	24		33.34 (0)	3	
Normalweigth	24.16 (38.3)	1329		32.39 (60.9)	486	
Overweigth	25.06 (41.4)	1138		30.94 (50.1)	823	
Obese	**28.65** (55.9) *	618	< 0.001^d^	**38.04** (59.1)^c^	356	0.22^d^
**Waist Circumference**			< 0.001			0.07
60–79 cm	23.31 (36.9)	834		**37.01** (119.9)	39	0.04^d^
80–87 cm	23.53 (41.1)	830		30.97 (49.5)	207	
88–94 cm	25.13 (36.5)	595		29.17 (45.5)	390	
95–101 cm	**28.94** (45.9) *	430	0.02^d^	**34.85** (60.5)^c^	431	0.43^d^
102–168 cm	**29.16** (58.8) *	447	0.003^d^	**34.11** (56.7)^c^	616	0.31^d^
**Smoking**			0.51			0.13
Yes	23.70 (38.9)	287		30.33 (45.5)	150	
No	24.57 (41.4)	1685		32.99 (52.6)	996	
Stopped Smoking	25.68 (43.5)	1048		**34.36** (62.1)^c^	465	
**Diagnosed hypertension**			< 0.001			0.002
Yes	**28.28** (56.4) *	636		**38.96** (70.4) *	377	
No	24.33 (39.4)	2416		31.83 (51.2)	1240	
**Diagnosed high Cholesterol**			0.003			0.007
Yes	**33.13** (57.8) *	254		**41.26** (81.4) *	227	
No	24.62 (40.7)	2798		32.12 (52.2)	1390	
**Physical activity during leisure time**			0.03			0.06
Non	**27.93** (47.7) *	297		27.72 (41.2)	192	
Little	24.23 (39.6)	2436		33.10 (57.4)	1187	
Regular	26.42 (64.3)	267		**34.22** (61.7)^c^	209	
**Family history of MI**			0.25			0.11
Not known	25.03 (40.4)	2803		32.36 (56.0)	1507	
Parental MI < 60	26.90 (59.0)	224		**43.20** (66.1)^C^	108	
**Family history of stroke**			0.98			0.68
Not known	25.15 (41.3)	2847		32.71 (53.7)	1523	
Parental Stroke < 60	24.78 (47.5)	187		**36.58** (76.5)^c^	90	
**Self-reported stress**			0.78			0.45
Never /sometimes stressed	25.05 (41.5)	2333		32.67 (54.2)	1356	
Always stressed	24.71 (42.4)	692		**34.18** (68.4)^c^	263	

* p-value calculated with non-parametric analyses

^a^IQR measures dispersion, between 25th percentile and 75th among HCC values in each variable

^b^The median in bold expresses the median above the general median for both sexes

^c^P-value where the median value is higher than the median for both sexes

**Table 5:** Cortisol levels and the odds ratios including 95% confidence intervals for being affected by previous cardiovascular diseases. Cortisol levels were divided into the highest decile (10%) compared to the lower decile (90%) in the total SCAPIS study population. Correlations were made with Spearman´s coefficients based on log-transformed cortisol values

Variables	HCC Highest Decile (*n* = 436)	HCC Medium and Lower deciles (*n* = 4385)	Univariate	Multivariate
	% *n*	% *n*	OR (CI 95%)	OR (CI 95%)
Myocardial infarction			2.73 (1.45,5.07) < 0.001	2.49 (1.33, 4.67) 0.005
Yes	3.1 13	1.2 49		
No	96.9 408	98.8 4199		
**Angina pectoris**			1.69 (0.65,4.38) 0.27	n/a
Yes	1.2 5	0.7 30		
No	98.8 416	4218 99.3		
**Atrial fibrillation**			2.62 (1.48, 4.67) < 0.001	2.25 (1.21, 4.17) 0.01
Yes	3.6 15	1.4 59		
No	96.4 406	98.6 4189		
**Heart failure**			3.18 (1.61, 8.72) 0.02	n/a
Yes	1.2 5	0.4 16		
No	98.8 416	99.6 4232		
**Bypass surgery or PCI**			2.44 (1.21, 4.89) 0.01	n/a
Yes	2.4 10	1.0 42		
No	97.6 411	99.0 4206		
**Stroke**			1.67 (0.82–3.40) 0.16	n/a
Yes	2.1 9	1.4 55		
No	97.9 412	98.7 4193		
**Type 2 Diabetes**			1.73 (1.14, 2.63) 0.01	1.71 1.11, 2.65) 0.02
Yes	6.4 27	3.8 162		
No	93.6 394	96.2 4086		
**Sleep apnea**			1.13 (0.68, 1.85) 0.64	n/a
Yes	4.3 18	3.8 162		
No	94.7 403	96.2 4086		

In the multivariate column, n/a indicates that the factor was not identified as statistically significant in a multivariate model discriminating those in the highest decile of hair cortisol from those in the lower decile

**Table 6:** Odds ratios and 95% confidence intervals for being affected by different cardiovascular risk factors. The highest decile (10%) compared to lower deciles (90%) in the total SCAPIS study population. Correlations were made with Spearman´s coefficients and logaritmized cortisol values

Variables	HCC Highest Decile (*n* = 436)	HCC Medium and Lower Decile (*n* = 4385)	Univariate	Multivariate
	% *n*	% *n*	OR (CI 95%) *p*-value	OR (CI 95%) *p*-value
**Fasting glucose mmol/l**			1.54 (1.18, 2.00) < 0.001	n/a
Over reference value	17.3 75	12.0 521		
Within reference value	82.7 359	88.0 3831		
**Diagnosed high cholesterol**			1.58 (1.18, 2.11) 0.002	n/a
Yes	14.7 62	9.9 419		
No	85.3 359	90.1 3829		
**Diagnosed hypertension**			1.50 (1.20, 1.87) < 0.001	1.49 (1.18, 1.87) 0.001
Yes	28.5 120	21.0 893		
No	71.5 301	79.0 3355		
**Leukocytes X10 ~ 9/L**			1.54 (1.11, 2.16) 0.01	1.47 (1.03, 2.10) 0.03
Over reference value	10.1 44	6.8 296		
Within reference value	89.9 392	93.2 4075		
**High-sensitivity CRP mg/L**			1.58 (1.12, 2.23) 0.009	1.50 (1.04, 2.17) 0.03
Over reference value	9.4 41	6.2 270		
Within reference value	90.6 395	93.8 4111		

In the multivariate column n/a indicates that the factor was not identified as statistically significant in a multivariate model discriminating those in the highest decile of hair cortisol from those in lower decile

### Ethics approval and consent to participate

The research protocol and all methods in the study were carried out in accordance with relevant guidelines and regulations. The SCAPIS study was approved by the Ethical Review Board (Dnr 2010-228-31 M). All participants gave their written informed consent to participate.

## Results

In this large population-based cohort, males had significantly (*p* < 0.001) higher HCC (median 32.6 mg/pg) than females (median 25.1 mg/pg). In the limited age span in this study (50–64 years), there was no significant difference in HCC with increasing age. Educational level and hair color were not associated with HCC. Individuals of both sexes born outside Europe and in other Nordic countries tend to have a higher median of HCC, especially females born in other Nordic countries, see Table 3.

Cardiovascular risk factors in relation to the median and Interquartile range of HCC for males and females is reported in Table 4.

Body mass index (BMI) was significantly (*p* < 0.001) associated with higher HCC for females, but not significantly for males. Both females (*p* < 0.001) and males (*p* = 0.22) classified as obese had the highest median of HCC. With increased waist circumference, the HCC values increased significantly among females although not significantly among men. Smoking was not significantly associated with HCC. Participants reported diagnosed hypertension had significantly higher HCC, both among females (*p* < 0.001) and males (*p* = 0.002). Those reporting diagnosed high cholesterol levels had also significantly higher HCC values for females (*p* = 0.003) and males (*p* = 0.007). Regarding physical activity during leisure time, those females who reported non-activity showed a significant (*p* = 0.03) increase in HCC, while the opposite was seen among males, those with regular activity tended to have (*p* = 0.06) higher HCC. No significant association was seen either for females or males for HCC in relation to self-reported stress.

The odds ratios of have been affected by previous cardiovascular diseases and chronic conditions within the highest deciles of HCC compared to the medium and lower deciles shown in Table 5.

Those in the highest HCC decile reported higher odds ratios for all studied cardiovascular diseases, significantly for myocardial infarction, atrial fibrillation, heart failure, bypass surgery or PCI, and type 2 diabetes. When corrected for confounders in multivariate analysis, the diagnosis of myocardial infarction, atrial fibrillation, and type 2 diabetes were identified as statistically independent discriminators of those in the highest versus lower deciles of HCC. The odds ratios of different cardiovascular risk factors are shown in Table 6.

All these factors (fasting glucose, cholesterol, hypertension, leukocytes, and hs-CRP above the reference value) were significantly and around 50% higher among those in the highest HCC deciles. As seen in the multivariate column of Table 6, hypertension, elevated leucocytes, and hs-CRP, were identified as statistically independent discriminators of those in the highest versus lower deciles of hair cortisol.

Of the discriminators identified as statistically independent in Tables 5 and 6, the cardiovascular diseases myocardial infarction OR = 2.49 (CI 1.33, 4.67), atrial fibrillation OR = 2.25 (CI 1.21, 4.17) and hypertension OR = 1.49 (CI 1.18, 1.87), hs-CRP OR = 1.50 (CI 1.04, 2.17) and the immune status of leucocytes OR = 1.47 (CI 1.03, 2.10) were all identified as statistically independent discriminators of those in the highest versus lower deciles of hair cortisol.

## Discussion

Increased hair cortisol concentrations (HCC) were in this study associated with various classical cardiovascular risk factors such as hypertension, high cholesterol, BMI and waist circumference (for women), type 2 diabetes, elevated fasting glucose, and high-sensitive CRP. Furthermore, increased HCC was associated with previous cardiovascular diseases like myocardial infarction. This study demonstrates that there is an association between the endocrine response and chronic stress that may affect the risk of cardiovascular risks and diseases [34, 35]. The cohort of middle-aged people from the general population analyzed in this study is probably one of the largest samples available with data on hair cortisol together with extensive data on cardiovascular risk factors through clinical investigations, blood samples, and questionnaires.

There were clear differences in HCC between males and females, with males demonstrating higher HCC than females, as found in previous studies [7, 10, 18, 21]. Although HCC tends to increase with aging [14], there were no significant age differences in HCC for this middle-aged cohort. This is in accordance with some other studies that did not find any age differences in HCC [7, 12]. There were no significant differences in hair cortisol concentrations by education which neither was found in the Whitehall 2 study [7].

Classical cardiovascular risk factors like hypertension, high cholesterol levels, and type 2 diabetes were all associated with HCC, but not smoking. Increased HCC was also associated with cardiovascular conditions including arterial fibrillation and bypass surgery. Other cardiovascular conditions like stroke, angina pectoris, heart valve disease, or sleep apnea were unrelated to HCC. Associations between HCC and cardiovascular diseases could also reflect a pathway where HCC is mediated by classical cardiovascular risk factors which has been shown [36]. Prospective studies are needed to further elucidate the relationship between elevated HCC and the risk for cardiovascular diseases. An association between HCC levels and values over the reference limits for blood glucose, hs-CRP, and leukocyte numbers was found. A previous study has documented an association between HCC, inflammation, and hs-CRP in connection with an analysis of cognitive functioning [37]. These findings indicate a linkage between HCC and inflammation and hypothetically also between HCC and the bodily immune defense. Cortisol is known to have an inhibitory effect on the immune system [38]. This association between the stress response and the immune response is a biomechanism that has previously been documented [39].

Physical activity and sports activities might increase the HCC values, but elevated hair cortisol concentrations often found among regular exercisers are not necessarily pathological [40]. HCC for this middle-aged normal population was significantly higher for females with less physical activity. Contrary HCC was slightly higher among males with regular and active physical activity. This gender-related difference might be explored in coming studies.

Cortisol in hair, a marker of long-term physiological and self-perceived stress, are often inconsistent and seldom exhibit any strong intercorrelations with each other [38]. In our study, like in most others, there was no significant association between self-reported stress and biologically measured stress by HCC [8, 9, 16].

Measuring HCC in larger populations means that some outliers also will appear. These could be of particular interest and could provide new and valuable information. However, all HCC values are log transformed to diminish their impact on the analysis. In this study, we found no significant differences in HCC in relation to hair color, which is also the finding in most other studies [4, 12], while some studies did find such associations [7]. In general, when discussing laboratory analysis, other techniques could also be useful discovering cardiovascular disorder or risk factors i.e. postmortem CIED (cardiac implantable electronic device) investigation could be an important tool in forensic medicine this tool also may unveil unsuspected, potentially lethal device malfunctions. An integrated approach between different working groups should be beneficial from patients’ point of view [41].

### Strength and limitations

The major strength of this study where we have measured cortisol concentrations in hair is its sample size and the population-based design. There are few similar studies of this magnitude except the Whitehall II study in the U.K [7]. Nevertheless, the sample size, of the study carries some risk of selection bias. We know from the SCAPIS pilot study that low socioeconomic status was associated with lower participation rates in the study [42]. Due to the study’s observational and cross-sectional nature, a general limitation is that we cannot establish causality.

A methodological issue when measuring cortisol in hair is that steroid medication might result in elevated measured cortisol levels. The studied cohort had a total of 100 participants who reported regular treatment with steroid ointments and creams, and they had significantly higher HCC. These participants were, therefore, omitted from further analysis to limit the potential influence of this factor.

A general limitation in studies measuring hair cortisol is that people, mainly males, with insufficient hair length cannot be included. This could have an impact on the results if a lack of hair or insufficient hair length is related to chronic stress. However, there is no evidence indicating that this might be a confounder. A previous analysis of the non-attendees (men without hair sample vs. men with hair sample) in the SCAPIS cohort showed that they did not differ from the participants concerning educational background or cardiovascular risks [18]. A limitation of the RIA method is that for around 2–3% of a population, it could give very high cortisol values, but the occurrence of these high values has not yet been explained [33]. This phenomenon entails that it is mainly possible to compare groups, and not traditional diagnostics to establish reference intervals. Since cortisol levels rise with age, women in this study who are all over 50 years of age, had entered menopause which may influence the cortisol levels and also the risk of cardiovascular diseases [34]. These potential factors have not been evaluated in this study, which is a limitation. Some participants reported unawareness of a family history of MI and Stroke, this memory bias might also have some impact and limit the interpretation of the results.

A minor limitation of this study is that we were not able to elucidate the potential impact on HCC of different types of hair treatments. Other studies have shown that dyed or colored hair and hair-washing frequency might influence the HCC [21]. However, these possible intervening factors have shown only to have marginal effects on our applied radioimmune assay method to measure HCC [18].

## Conclusions

In conclusion, long-term stress, measured by hair cortisol, was significantly associated with the major cardiovascular risk factors hypertension and high cholesterol, but not smoking. Those with elevated HCC levels also had a significantly increased pre-history of myocardial infarction, type 2 diabetes, atrial fibrillation and by-pass surgery, but not regarding stroke, angina pectoris or sleep apnoea. Furthermore, HCC was associated with risk markers like, leukocytes, and high-sensitivity CRP, indicating a possible linkage between HCC and inflammation and hypothetically also the bodily immune defense. No association was found between perceived stress and HCC. Altogether, the analyses of HCC in a large middle-aged population show that chronically elevated cortisol levels represent a relevant and significant factor associated with cardiovascular diseases and classical cardiovascular risk factors. An overall conclusion of our results is that health care should put more emphasis on patients reporting that they have been exposed to long term stress.

## Data Availability

The data that support the findings of this study are available on request from the corresponding author: ashild.olsen.faresjo@liu.se The data are not publicly available due to privacy or ethical restrictions and are stored at the repository at Linköping University Electronic Press https://ep.liu.se.
